# Unveiling the Mechanisms Underlying the Immunotherapeutic Potential of Gene–miRNA and Drugs in Head and Neck Cancer

**DOI:** 10.3390/ph17070921

**Published:** 2024-07-10

**Authors:** Danishuddin, Md Azizul Haque, Md. Zubbair Malik, Rakesh Arya, Pooja Singh, Jeong-Sang Lee, Jong-Joo Kim, Keun-Woo Lee, Tae-Sung Jung

**Affiliations:** 1 Department of Biotechnology, Yeungnam University, Gyeongsan 38541, Republic of Korea; danish23@yu.ac.kr (D.); azizul@ynu.ac.kr (M.A.H.); rakesharya101@yu.ac.kr (R.A.); 2 Department of Genetics and Bioinformatics, Dasman Diabetes Institute (DDI), Dasman 15462, Kuwait; zubbairmalik@jnu.ac.in; 3 Division of Applied Life Science (BK21 Four), Plant Molecular Biology and Biotechnology Research Center (PMBBRC), Gyeongsang National University (GNU), Jinju 52828, Republic of Korea; singhpooja@gnu.ac.kr; 4 GSCRO, Research Spin-Off Company, Innopolis Jeonbuk, Jeonju 55069, Republic of Korea; jslee11@jj.ac.kr; 5 Department of Food and Nutrition, College of Medical Science, Jeonju University, Jeonju 55069, Republic of Korea; 6 Korea Quantum Computing (KQC), Busan 48058, Republic of Korea; 7 Angel i-Drug Design (AiDD), Jinju 52650, Republic of Korea; 8 Laboratory of Aquatic Animal Diseases, Research Institute of Natural Science, College of Veterinary Medicine, Gyeongsang National University (GNU), Jinju 52828, Republic of Korea

**Keywords:** head and neck cancer, pathways, miRNAs, drugs, network biology

## Abstract

Head and neck cancer ranks as the sixth-most common malignancy worldwide, characterized by high mortality and recurrence rates. Research studies indicate that molecular diagnostics play a crucial role in the early detection and prognostic evaluation of these diseases. This study aimed to identify potential biomarkers for head and neck cancer and elucidate their interactions with miRNAs and possible therapeutic drugs. Four drivers, namely, *FN1*, *IL1A*, *COL1A1*, and *MMP9*, were identified using network biology and machine learning approaches. Gene set variation analysis (GSVA) showed that these genes were significantly involved in different biological processes and pathways, including coagulation, UV-response-down, apoptosis, NOTCH signaling, Wnt-beta catenin, and other signal pathways. The diagnostic value of these hub genes was validated using receiver operating characteristic (ROC) curves. The top interactive miRNAs, including *miR-128-3p*, *miR-218-5p*, *miR-214-3p*, *miR-124-3p*, *miR-129-2-3p*, and *miR-1-3p*, targeted the key genes. Furthermore, the interaction between the key genes and drugs was also identified. In summary, the key genes and miRNAs or drugs reported in this study might provide valuable information for potential biomarkers to increase the prognosis and diagnosis of head and neck cancer.

## 1. Introduction

Head and neck squamous cell carcinoma (HNSCC) comprises a group of tumors originating from the squamous epithelium in the oral cavity, oropharynx, larynx, and hypopharynx [1,2,3]. HNSCC is one of the most prevalent malignancies, ranking as the sixth-most common cancer worldwide [4,5]. The lungs are the most common site for distant metastasis in HNSCC, representing 70–85% of cases [6]. Over the past decades, several major pathogenic factors of HNSCC have been identified. The PI3K/Akt/mTOR pathway interacts with and plays a role in regulating several additional signaling molecules in HNSCC [7]. Additionally, the cyclooxygenase-2 (COX-2) signaling pathway is strongly linked to tumor angiogenesis in HNSCC, with COX-2 overexpression indicating a poorer prognosis for patients with head and neck cancer [8]. Among these, HPV has emerged as the most well-studied and frequently utilized biomarker in HNSCC [9,10,11]. While many factors have been identified as contributors to HNSCC, the precise pathogenic mechanisms of this disease are not yet fully understood. Clarifying these mechanisms is essential for identifying potential target genes for HNSCC treatment. Currently, the treatment options for managing head and neck cancer include radiation therapy, surgery, and chemotherapy. The appropriate combination of these modalities is selected based on the cancer’s location and stage [12,13]. Despite the availability of various treatments, patients with HNSCC still face limited survival benefits [14].

In recent years, bioinformatics analysis has gained significant prominence as a research hotspot due to its potential to revolutionize our understanding of complex biological systems. Among the various techniques in this field, the network biology approach stands out as a powerful method for analyzing differential gene expression [15,16]. This approach not only facilitates the study of molecular mechanisms underlying genome regulation but also helps in identifying key differences in gene expression between distinct biological states or conditions. By mapping the interactions and relationships between genes, network biology provides a comprehensive view of the biological networks that govern cellular functions. This holistic perspective is crucial for pinpointing key genes, also known as hub genes, which play central roles in these networks [17,18]. Identifying these hub genes is particularly valuable because they are often critical regulators of biological processes and can serve as potential biomarkers for various diseases.

In this study, the gene expression profile of head and neck cancer was downloaded from the TCGA database [19]. Differentially expressed genes (DEGs) were initially screened based on the dataset mentioned above. Subsequently, we used a comprehensive network biology approach to identify the key regulators for HNSCC. The interactions between key genes and miRNAs were then analyzed, followed by functional enrichment analysis to construct the regulatory networks of miRNAs and genes. Additionally, a gene–drug interaction analysis was also conducted to identify potential therapeutic drug candidates targeting the key regulators. In summary, the integration of network biology and differential gene expression analysis enhances our ability to identify crucial genes that may serve as potential biomarkers for HNSCC. This integrated approach not only deepens our understanding of the molecular mechanisms driving the disease but also opens new avenues for developing precision medicine strategies tailored to individual patients’ genetic profiles.

## 2. Results

**Figure 1:**
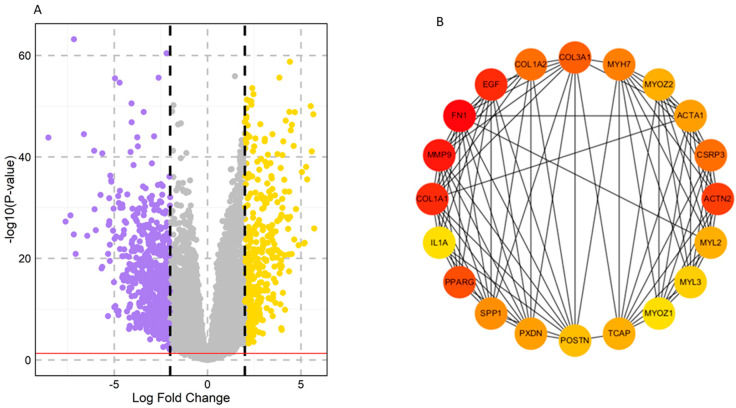
Screening and identification of key genes in the TCGA–HNSCC dataset. (**A**) Volcano plots of DEGs based on the TCGA–HNSCC dataset. Yellow color: upregulated, Violet color: downregulated, Grey color: non–significant. (**B**) Top 20 hub genes selected based on degree using the CytoHubba app in Cytoscape. The color represents the degree of interaction. The higher the ranking, the more intense the red color used, whereas orange and yellow colors represent an intermediate degree of interaction and a lower degree of interaction, respectively.

A total of 1065 DEGs were differentially expressed between the HNSCC and normal samples based on the criterion of *p* < 0.05. Among these, 405 genes were upregulated, and 660 genes were downregulated. The volcano plot and clustering module of DEGs are shown in Figure 1. To further investigate the functions and pathways of the DEGs, enrichment analysis was used, revealing the roles of these candidate genes.

### 2.1. Identification of Key Regulators or Hub Genes

**Figure 2:**
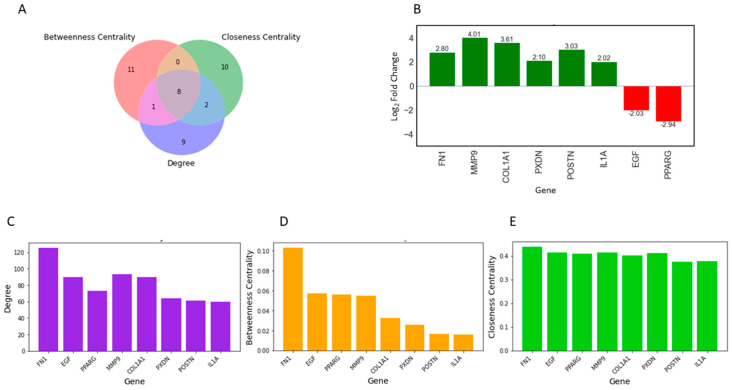
Identification of key regulators using three attributes of the PPI Network. (**A**) Intersections among top genes with the highest centrality values of closeness, betweenness, and degree. All up, 8 common genes were identified among the top 20 genes for each topological parameter. (**B**) Bar plot showing the fold change in expression of the 8 key regulators in individual healthy controls and HNSCC patients. The red bars represent the log_2_-fold change of upregulated genes, while the green bars represent the log_2_-fold change of downregulated genes. (**C**–**E**) The top 20 differentially expressed genes (DEGs) were assessed for their topological properties using centrality measurements, degree, betweenness, and closeness, in the network.

**Figure 3:**
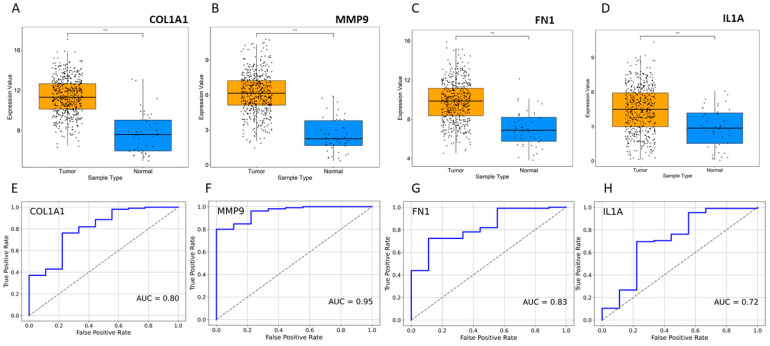
Expression profile of HNSCC. (**A**–**D**) Expression of four selected genes in disease vs. normal samples. *** *p* ≤ 0.001. (**E**–**H**) ROC curve for significant gene expression data. The AUC values indicate that patient groups with differing diseases and controls may be distinguished by the expression analysis of the markers.

Topological properties determine the structural characteristics of a complex biological network. Therefore, to understand the fundamental behavior of the PPI network, we analyzed its topological properties using the Network Analyzer and CytoHubba plugins in Cytoscape. In this context, the hubs of the network, which are the nodes with the highest degree, usually play a crucial role in the PPI network. Genes commonly predicted in the top 20 from the degree, betweenness centrality, and closeness centrality were identified as potential biomarkers. Ultimately, eight genes met our screening threshold consistently, including *COL1A1*, *IL1A*, *MMP9*, *PPARG*, *FN1*, *EGF*, *POSTN*, and *PXDN* (Figure 2A,B). Six out of the eight genes were found to be upregulated, with *MMP9* exhibiting the maximum fold change of 4.01, followed by *COL1A1* with a fold change of 3.62, while two genes were downregulated, with *PPARG* showing a fold change of −2.94 and EGF a fold change of −2.03 (Figure 2B). Furthermore, the expression of these four genes (*COL1A1*, *IL1A*, *MMP9*, and *FN1*) was significantly higher in HNSCC patients compared to normal samples (Figure 3A–D). Receiver operating characteristic (ROC) curve analyses were performed to assess the importance of key genes in HNSCC diagnosis and prognosis. The ROC analysis revealed that the mRNA expression levels of these four genes showed excellent diagnostic value for HNSCC cancer and adjacent tissues. The ROC curve analysis indicated that these four hub genes reasonably discriminated between HNSCC and normal samples (Figure 3E–H). Compared to normal samples, *MMP9*, with an AUC value of 0.95 (Figure 3F), had the largest area under the ROC curve, followed by *FN1* (Figure 3G). The AUC values suggest that these genes can distinguish between HNSCC and normal samples.

### 2.2. Functional Enrichment Analysis of DEGs

**Figure 4:**
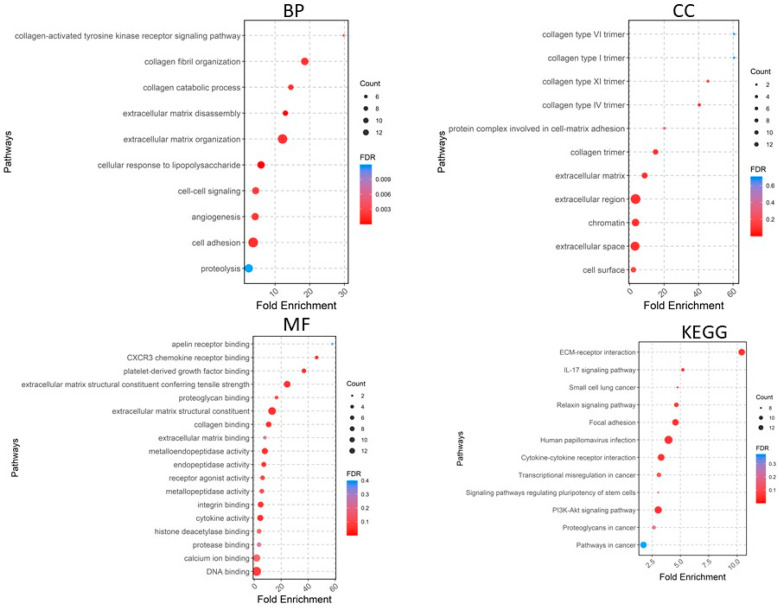
Gene Ontology analyses of upregulated genes. BP, biological process; CC, cellular component; MF, molecular function; KEGG, Kyoto Encyclopedia of Genes and Genomes.

**Figure 5:**
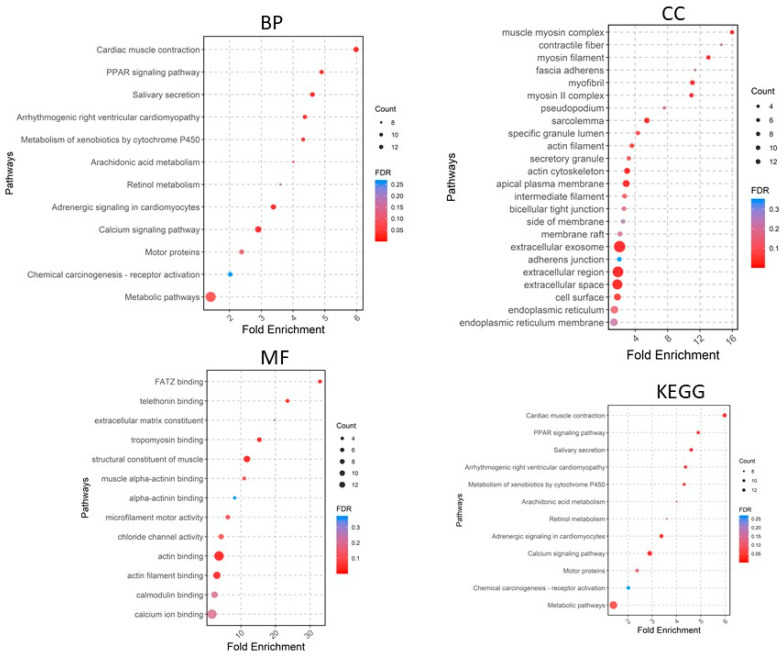
Gene Ontology analyses of downregulated genes. BP, biological process; CC, cellular component; MF, molecular function; KEGG, Kyoto Encyclopedia of Genes and Genomes.

A total of 405 upregulated genes and 660 downregulated genes were analyzed using the DAVID software version v2023q4 [20]. Gene Ontology (GO) enrichment analysis showed that in the Biological Process (BP) category, the upregulated DEGs were involved in extracellular matrix organization, collagen fibril organization, cell adhesion, angiogenesis, cell–cell signaling, etc. (Figure 4), whereas the downregulated DEGs were significantly associated with cardiac muscle contraction and more (Figure 5). For the CC category, the upregulated DEGs were correlated with the extracellular region, extracellular space, extracellular matrix, chromatin, etc. (Figure 4), whereas the downregulated DEGs were associated with the extracellular exosome, extracellular region, extracellular space, myofibril, etc. In the MF category, the upregulated DEGs were enriched in extracellular matrix structural constituent, metalloendopeptidase activity, platelet-derived growth factor binding, collagen binding, cytokine activity, integrin binding, and CXCR chemokine receptor binding (Figure 4), whereas the downregulated DEGs were related to structural constituent of muscle, actin binding, tropomyosin binding, FATZ binding, actin filament binding, etc. For KEGG pathway enrichment analysis, the upregulated pathways included ECM–receptor interaction, focal adhesion, *PI3K-Akt* signaling pathway, cytokine–cytokine receptor interaction, and relaxin signaling pathway (Figure 5), whereas the downregulated KEGG pathways included *PPAR* signaling pathway, cardiac muscle contraction, calcium signaling pathway, motor proteins, etc.

### 2.3. Relationship of Hub Genes and Disease-Related Genes

**Figure 6:**
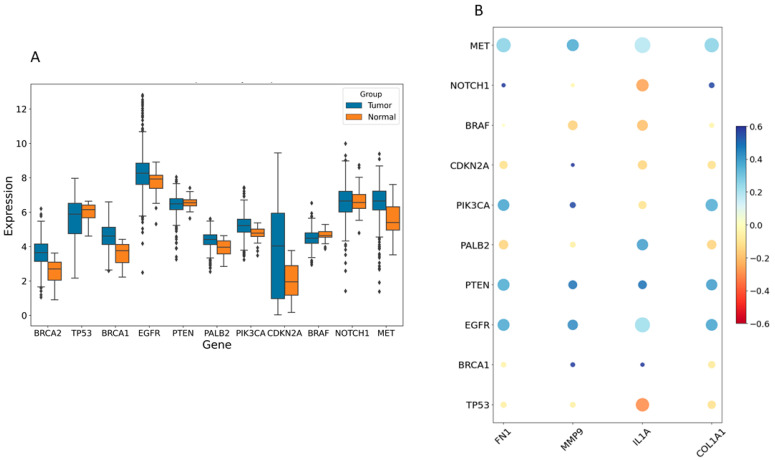
The relationship between hub genes and disease-related genes. (**A**) The expression levels of various disease-related genes were compared between control and individuals with HNSCC. (**B**) A bubble plot was used to visualize the Pearson correlations between four hub genes (*FN1*, *IL1A*, *MMP9*, and *COL1A1*) and disease-related genes. Larger circles indicate *p*-values closer to zero, while redder shades represent stronger negative correlations and deeper blue shades indicate stronger positive correlations.

The disease genes linked to HNSCC tumorigenesis were identified from the GeneCards database (https://www.genecards.org/, accessed on 9 May 2024). This analysis highlighted significant differences in expression levels of several disease-associated genes between the control and HNSCC groups, including *TP53*, *MET*, *NOTCH1*, *PTEN*, *PIK3CA*, *EGFR*, *CDKN2A*, *BRCA1*, *BRCA2*, *BRAF*, and *PALB2* (Figure 6A). Pearson correlation analysis revealed that *FN1*, *MMP9*, *COL1A1*, and *IL1A* were notably associated with the expression of these disease-related genes. Figure 6B illustrates that high expression of *COL1A1*, *MMP9*, and *FN1* positively correlated with *MET*, *EGFR*, *PTEN*, and *PIK3CA* (excluding *IL1A*) and negatively correlated with *TP53* and *CDKN2A* (excluding *MMP9*).

### 2.4. Gene Set Variation Analysis (GSVA) Analyses

**Figure 7:**
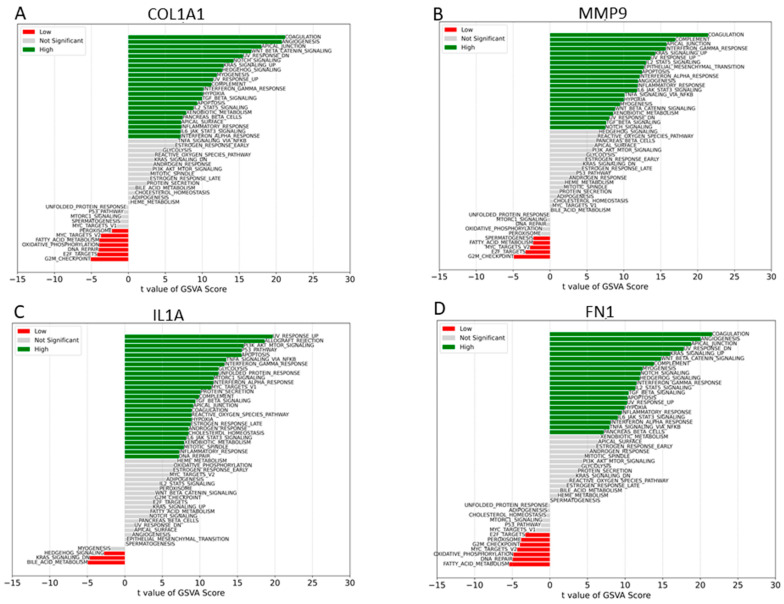
GSVA analysis of high and low expression. (**A**) GSVA of *COL1A1*. (**B**) GSVA of *MMP9*. (**C**) GSVA of *IL1A*. (**D**) GSVA of *FN1*.

We proceeded to analyze the specific signaling pathways involved in the four hub genes and explored the effect of candidate genes on the signaling pathways related to disease progression. GSVA results showed that high expression of *COL1A1* primarily enriched coagulation, UV-response-down, apoptosis, NOTCH signaling, Wnt-beta catenin, and other signal pathways (Figure 7). High expression of *FN1* mainly enriched signaling pathways such as UV-response-up, NOTCH signaling, interferon gamma response, TGF-beta signaling, *IL6-JAK-STAT3* signaling, and apoptosis, whereas *MMP9* is primarily enriched in signaling pathways such as coagulation, apical junction, UV response-up, KRAS signaling-up, interferon gamma response, and *IL6-JAK-STAT3* (Figure 7). Low expression of *IL1A* enriched KRAS signaling-down and bile–acid metabolism, whereas low expression of *MMP9* and *COL1A1* enriched DNA repair, E2F target, fatty acid metabolism, MYC target V2, and G2M checkpoint.

### 2.5. Prediction of miRNAs of Hub Genes and Enrichment Analyses

**Figure 8:**
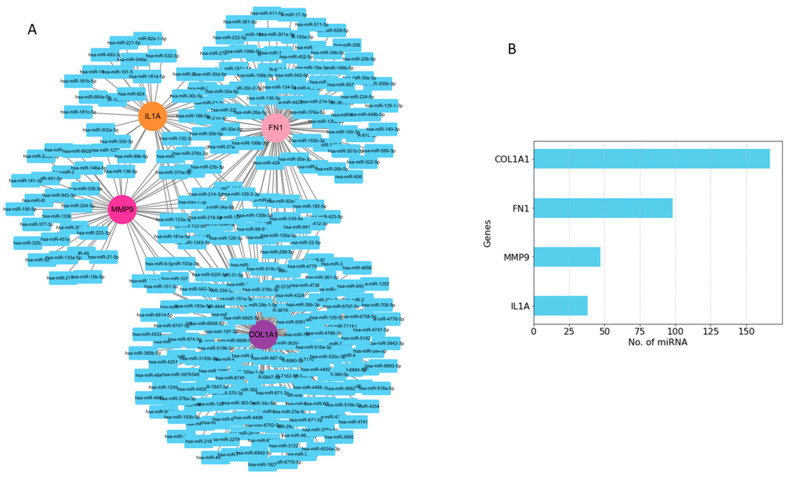
Predicted regulatory networks of selected potential genes and their associated miRNAs. (**A**) miRNAs related to *COL1A1*, *IL1A*, *FN1*, and *MMP9* genes were identified from the miRNet database. (**B**) The bar graph shows the number of predicted miRNAs.

**Figure 9:**
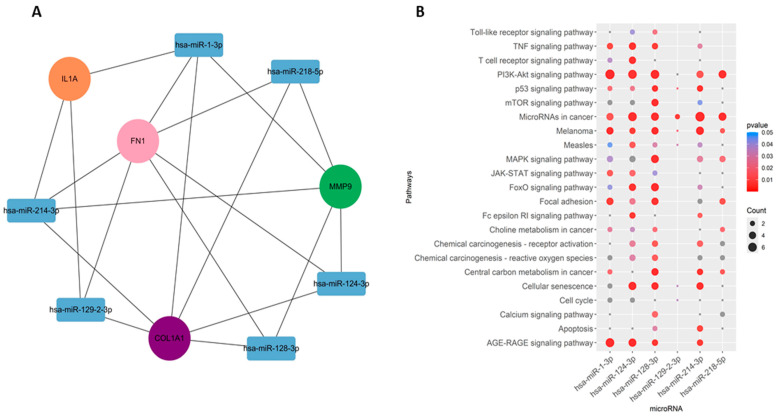
miRNA–gene regulatory network. (**A**) Regulatory network of key genes–miRNAs. (**B**) Pathways associated with the miRNAs.

Among the interacting miRNA–mRNA pairs, four genes (*COL1A1*, *IL1A*, *MMP9*, and *FN1*) were mapped to miRNAs using the miRNet database (Figure 8A). A significant number of miRNAs were mapped to each of these four hub genes (Figure 8B). *COL1A1* targets 168 miRNAs, *MMP9* targets 47 miRNAs, and *IL1A* targets 38 miRNAs, whereas *FN1* targets 98 miRNAs. Next, we embarked on constructing a key gene–miRNA network. Degree analysis of the key miRNA–mRNA regulatory network was used to identify the key miRNAs. We found that six of the above miRNAs, including *miR-1-3p*, *miR-218-5p*, *miR-124-3p*, *miR-128-3p*, *miR-214-3p*, and *miR-129-2-3p*, emerged as key miRNAs targeting four driver genes (*COL1A1*, *FN1*, *MMP9*, and *IL1A*) (Figure 9A). Functional enrichment analysis was performed on the predicted miRNAs from the key hub gene–miRNA analysis. Biological pathways and KEGG pathway enrichment analysis were successively identified by MIENTURNET to validate these miRNAs. Enrichment analysis revealed that these miRNAs were mainly enriched in *p53* signaling, *mTOR*, cell cycle, focal adhesion, and *PI3K-AKT* and *MAPK* signaling (Figure 9B).

### 2.6. Drug–Gene Interaction Analysis

**Figure 10:**
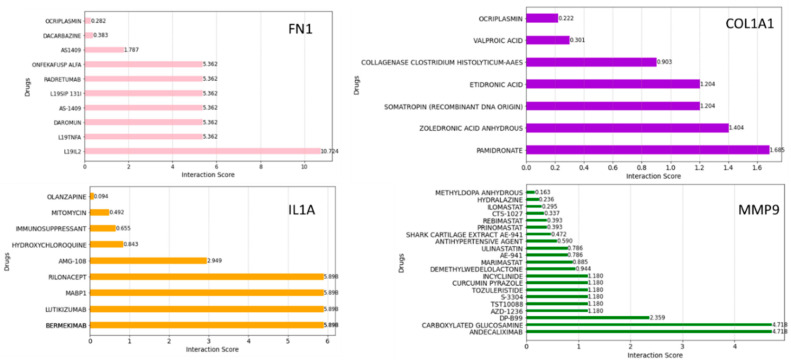
Drug–gene interaction analyses using the DGIdb. The bar plot shows the possible therapeutic drugs significant for HNSCC in terms of interaction scores.

To further refine our search, we employed the Drug–Gene Interaction Database (DGIdb) to identify compounds with high interaction scores with the hub genes. Figure 10 shows the drugs related to *FN1*, *COL1A1*, *IL1A*, and *MMP9*. These drugs were ranked based on the highest total score in the Drug–Gene Interaction Database. They may hold promise as potential therapeutic agents, which could aid in the development of new treatment targets for HNSCC cancer therapy; however, further investigation is needed to determine their efficacy and safety.

## 3. Discussion

### 3.1. Key Regulators Gene Analyses

In our investigation, we delved into the potential biomarkers and mechanisms underlying HNSCC tumors through systematic bioinformatics analysis. We conducted a comprehensive differential gene expression analysis (DGEA) between HNSCC cancer and normal samples sourced from the TCGA database. Our analysis identified a total of 405 upregulated and 660 downregulated genes. GO and KEGG pathway enrichment analyses indicated that upregulated genes were mainly enriched in extracellular matrix organization, collagen fibril organization, cell adhesion, endodermal cell differentiation, angiogenesis, and cell–cell signaling. Using the consensus network biology approach, we identified four key genes, including *COL1A1*, *IL1A*, *MMP9*, and *FN1*. *COL1A1*, which encodes collagen type I alpha 1 chain, has been implicated in promoting metastasis in various cancers. Collagen, the main component of ECM, is an essential component for normal tissue function; it has crucial roles in maintaining stability and integrity in both tissues and organs [21]. *COL1A1* can enhance the activity of MMPs, particularly *MMP2*, which degrade the ECM and facilitate tumor cell invasion and metastasis [22,23]. However, its role in head and neck cancer is not fully understood, and further research is needed to elucidate the specific mechanisms by which *COL1A1* contributes to tumor progression and metastasis in this context. Matrix metalloproteinases (MMPs) are a family of zinc-dependent proteases that play crucial roles in remodeling the extracellular matrix (ECM) and basement membranes, as well as regulating growth factors, cytokines, and chemokines [24]. One member, *MMP9*, is an inducible protease expressed by tumor epithelial cells. It is associated with macrophage and neutrophil infiltration and regulates ECM remodeling, neovascularization, and inflammatory signaling [25]. Matrix metalloproteinases (MMPs) emerged as key players, particularly *MMP9*, whose overexpression is implicated in HNSCC invasion and metastasis [26]. Iron-induced *MMP9* expression through the activation of AP-1 via the *ERK/AKT* pathway underscores the intricate regulatory mechanisms governing HNSCC progression. Additionally, transforming growth factor-beta1 (TGF-β1) was identified as a potential regulator of *MMP9* expression, further highlighting the complex signaling networks contributing to HNSCC pathogenesis [27]. *FN1* is significantly overexpressed in HNSCC patients and has been associated with higher pathological stages and poor prognosis [28]. *FN1* activation contributes to tumor growth and progression by stimulating *PI3K-AKT* signaling, leading to enhanced cell survival, proliferation, and metastasis [28]. The *PI3K-AKT* signaling pathway is reported to be the most frequently altered oncogenic pathway and is genetically altered in most HNSCC tumors [29]. The *PI3K-AKT* pathway is a crucial signaling cascade involved in cell growth, proliferation, survival, and metabolism [30]. Interleukin-1 alpha (*IL1A*) plays a significant role in head and neck cancer by primarily acting through the NF-κB signaling pathway. NF-κB is a transcription factor involved in regulating various cellular processes, including inflammation, immune responses, cell proliferation, and survival. In HNSCC, *IL1A* expression is often upregulated, leading to the activation of NF-κB signaling [31]. This activation promotes tumor growth, invasion, metastasis, and resistance to apoptosis. Additionally, *IL1A*-*NF-κB* signaling induces the secretion of pro-inflammatory cytokines and chemokines, creating a tumor-promoting microenvironment. Therefore, *IL1A*’s involvement in HNSCC pathogenesis highlights its potential as a therapeutic target for inhibiting tumor progression and improving patient outcomes.

### 3.2. GSVA Analyses

The GSVA analysis indicated that different expression levels of *IL1A*, *COL1A1*, *MMP9*, and *FN1* might influence various signaling pathways related to disease progression, including coagulation, angiogenesis, apical junction, Wnt signaling pathways, and NOTCH signaling. Angiogenesis is widely acknowledged for its involvement in the progression of head and neck cancer, as well as its role in conferring resistance to drugs and radiotherapy. It is essential for the growth and progression of head and neck tumors by providing oxygen and nutrients to the growing cancer cells [32]. *COL1A1* expression may promote angiogenesis by modulating the ECM composition and promoting the secretion of angiogenic factors, thereby supporting tumor growth and metastasis [33]. However, despite the importance of angiogenesis in head and neck cancer, only a small subset of antiangiogenic agents has proven effective in clinical practice and received approval for treating this condition [33]. Several other studies have shown that abnormal activation of the Wnt signaling pathway facilitates tumor transformation in head and neck tissues [34]. The NOTCH signaling cascade plays a vital role in various cellular processes, including cell proliferation, differentiation, and survival [35]. Dysregulation of the NOTCH pathway is linked to the progression of numerous malignant tumors, such as head and neck squamous cell carcinoma. Apical junction pathways play crucial roles in maintaining the integrity and function of epithelial tissues, which are often disrupted in cancer [36]. Epithelial cells form barriers between different tissue compartments and are held together by various junctional complexes, including tight junctions, adherens junctions, and desmosomes, collectively known as apical junctions.

### 3.3. Gene–Disease-Related Gene Interaction Analyses

Furthermore, our investigation unveiled correlations between these four hub genes and the expression of numerous disease-related genes, including *MET*, *BRAF*, *PTEN*, *EGFR*, and *PIK3CA*. Our data demonstrate that *COL1A1*, *IL1A*, *MMP9*, and *FN1* are positively correlated with *MET*, *PTEN*, *EGFR*, and *PIK3CA*. This correlation suggests a complex interplay between these genes and pathways, influencing tumor progression and therapeutic responses in HNSCC. Head and neck cancers often have genetic mutations that activate oncogenes like *PIK3CA* or inactivate the *PTEN* tumor suppressor gene. Such changes lead to the increased activity of a key growth and survival pathway (*PI3K/AKT/mTOR*), which is commonly seen in head and neck squamous cell carcinomas. The epidermal growth factor receptor (*EGFR*), part of the ErbB family of receptor tyrosine kinases (RTKs), is recognized as a potential therapeutic target for HNSCC. Multiple agents targeting *EGFR* have been created and examined in clinical trials for HNSCC [37,38]. Understanding the relationship between *FN1* and *COL1A1* and key genes like *PTEN*, *EGFR*, and *PIK3CA* can have implications for therapeutic strategies in controlling HNSCC. Targeting *FN1*, *COL1A1*, or downstream signaling pathways may offer new therapeutic opportunities for inhibiting tumor growth and improving treatment responses. Cox et al. showed that co-expression of *MMP9* and *EGFR* confers a poor prognosis in non-small cell lung cancer [39]. They proposed that the *EGFR* signaling pathway might contribute significantly to the invasive tendencies of NSCLC through the upregulation of *MMP9* [25]. c-*MET* is often aberrantly activated in cancer, leading to the persistent activation of several downstream signaling pathways, including the mitogen-activated protein kinase (MAPK) and phosphatidylinositol 3-kinase (*PI3K*)-*AKT* pathways [29]. In summary, our findings shed light on the intricate molecular landscape of HNSCC, underscoring the potential of identified biomarkers and pathways as promising targets for therapeutic intervention and further exploration.

### 3.4. Gene–miRNAs Interaction Analyses

MicroRNAs (miRNAs), which are small non-coding RNAs, play crucial roles in gene regulation and cancer biology [40]. These miRNAs can bind to mRNA and control their expression, thereby influencing various cellular processes [40,41,42,43]. In order to comprehensively recognize the role and potential mechanism of the *COL1A1*, *IL1A*, *MMP9*, and *FN1* genes in the prognosis of head and neck cancer, we constructed a miRNA-related regulatory network in the current study. Firstly, we extracted the mRNA–miRNA relationship pairs related to the mRNAs of the four hub genes from the miRNet database, resulting in a total of approximately 202 miRNAs (Figure 6). The constructed regulatory networks of miRNA–mRNA (target genes) revealed six key miRNAs, namely, *miR-128-3p*, * miR-218-5p*, * miR-214-3p*, * miR-124-3p*, *miR-129-2-3p*, and *miR-1-3p*. These miRNAs corresponded to the top nodes with the highest degree in the constructed network and have shown involvement in head and neck cancer as well as other cancers.

*miR-1-3p* can act as a tumor suppressor or promoter, depending on the specific cellular context and target genes involved [40,44]. It regulates cell proliferation, apoptosis, invasion, and metastasis by targeting oncogenes or tumor suppressor genes, thereby influencing cancer progression and response to therapy. A study has shown that low expression of miR-1-3p may facilitate tumorigenesis and evolution in HNSCC through signaling pathways [45]. miR-124-3p regulates cancer progression pathways, including *PI3K/AKT*, *Wnt/β-catenin*, and *MAPK*, by targeting key components to modulate proliferation, survival, and invasion in HNC cells. It acts as a tumor suppressor by directly interacting with PCDH8 and inhibiting the *PI3K/AKT/mTOR* signaling pathway [46]. Another study has shown that *miR124-3p* and *miR766-3p* are overexpressed in resistant HNSCC, correlating with poor prognosis and contributing to the acquisition of resistance phenotype [47]. In vitro and in vivo studies have shown that elevated levels of *miR766-3p* and *miR124-3p* promote aggressive HNSCC behavior, including drug resistance, proliferation, and invasion. *miR-129-2-3p* has been identified for its inhibitory effects on multiple cancer types; however, its impact on the growth and invasion of head and neck cancer has not been fully understood and extensively investigated.

The overexpression of *miR-128* suppresses the development of HNSCC by directly targeting Paip-interacting protein 2 (*Paip2*), BAG Cochaperone 2 (*BAG-2*), H3F3B, Bmi-1, and Bcl-2-associated X protein, which are involved in proliferative and apoptotic processes. Upregulated *miR-128* expression inhibits HNSCC cell growth and promotes apoptosis in HNSCC cells, partially through its direct modulation of these targets [48]. In our analyses, the hub genes identified in this study were found to be the targets of these miRNAs; however, verification through in vitro experiments is needed. Currently, many aspects of the mechanism of miRNA regulating hub genes in HCC are still unknown.

### 3.5. Gene–Drugs Interaction Analyses

DGIdb is a curated database of drug–gene interactions mined from various sources such as DrugBank, PharmGKB, ChEMBL, Drug Target Commons, and others. Using the DGIdb database, a total of 10 drugs for each hub gene were identified. Dacarbazine has been the most effective single agent for treating advanced metastatic melanoma and has remained the standard chemotherapy for this malignancy for over 30 years [49]. In our study, dacarbazine interacted with *FN1*, suggesting that *FN1* might be a potential drug target for dacarbazine in anti-HNSCC therapy. Mitomycin is an older chemotherapy drug that has been in use for decades. As an antibiotic, it has demonstrated antitumor activity by selectively inhibiting the synthesis of DNA [50]. In our study, mitomycin interacted with *IL1A*. Andecaliximab (ADX) is a monoclonal antibody that inhibits matrix metalloproteinase 9, an extracellular enzyme involved in matrix remodeling, tumor growth, and metastasis [51]. A phase I and Ib study of modified oxaliplatin, leucovorin, and fluorouracil (mFOLFOX6) combined with ADX demonstrated encouraging antitumor activity in patients with gastric or gastroesophageal junction (GEJ) adenocarcinoma. Andecaliximab was found to interact with *MMP9* with a high interaction score. The pharmacological mechanisms between the hub genes and drugs need to be further explored.

### 3.6. Advantages, Medical Applications, and Limitations of this Study

The advantage of our study is that it integrates complex data from curated databases like TCGA and DGIdb with network biology. Network biology considers biomarkers within the broader context of biological networks, such as protein–protein interaction networks, gene regulatory networks, or metabolic networks. This context allows for a deeper understanding of how biomarkers are interconnected and function within biological pathways. Our approach offers insights into disease mechanisms and potential therapeutic targets. By identifying hub genes, we pave the way for personalized medicine strategies that could enhance diagnosis, prognosis, and treatment outcomes. The miRNAs and drugs associated with hub genes hold promise for targeted therapies. For example, leveraging these findings could lead to more effective treatments tailored to individual genetic profiles, potentially improving patient outcomes. Despite its advantages, our study has some limitations. The principal limitation of our study is the lack of independent experimental validation. Although four hub genes were identified, their role in HNSCC pathogenesis lacks substantial validation, and their underlying mechanisms of action remain unclear. We acknowledge the need for rigorous experimental validation of predicted interactions and effects. Furthermore, while promising, clinical translation would require robust clinical trials to establish efficacy and safety.

## 4. Material and Methods

### 4.1. Dataset

The dataset related to HNSCC was downloaded from TCGA (https://portal.gdc.cancer.gov/, accessed on 5 April 2024) [19]. Screening for differentially expressed genes (DEGs) between cancer and normal samples was performed using the R package Limma [52] and BIOMAX [53]. The DEG threshold was set at an adjusted *p* < 0.05 and a log_2_-fold change > 2. The identified upregulated and downregulated genes were subsequently used for pathway analysis and construction of the PPI Network.

### 4.2. Functional Enrichment Analysis of the DEGs

To investigate the enrichment of biological functions among the DEGs, both the upregulated and downregulated DEGs were analyzed using the Database for Annotation, Visualization, and Integrated Discovery (DAVID) [20]. Gene Ontology (GO) analysis [54] and Kyoto Encyclopedia of Genes and Genomes (KEGG) pathway analysis [55] were conducted on specific genes. A significance level of *p* ≤ 0.001 was considered statistically significant.

### 4.3. Key Regulators (KR)

To understand the regulatory function of genes, a protein–protein interaction (PPI) network was constructed using the STRING database [56]. Physical interaction data from the STRING database were utilized for constructing, visualizing, and analyzing networks on the Cytoscape v3.6.0 platform [57]. The topological properties of the network were analyzed using the Network Analyzer [58] and CytoHubba [59] plugins of Cytoscape. The most impactful nodes within the network are recognized as its hubs. Nodes with the highest degrees and corresponding high centrality values were pinpointed as hubs within the PPI network. To identify the key regulators (KRs) within the PPI network, the initial step involved identifying the network’s crucial genes based on their degrees (k) and centrality measures (Cc, C_B_). The top 20 genes with different topological properties were compared to identify KR genes.

### 4.4. Topological Properties of the Network

The fundamental topology of a network is typically established by examining its key topological parameters, namely, P(k), C(k), and CN(k) [58]. The topology of the gene network was determined by analyzing these three parameters using the Network Analyzer version 4.5.0 [58] in Cytoscape v3.6.0 [57], with the network treated as undirected. The values of the three parameters P(k), C(k), and CN(k) were plotted against the degree k and their distribution patterns were assessed to characterize the network’s topological features.

### 4.5. Degree

The degree of a node represents the total number of connections it has within the network, denoted by k. This metric is considered a measure of the node’s local importance in network regulation. The graph is represented as G = (N, E), where E represents the edges and N represents the nodes. C_B_ and C_C_ serve as fundamental parameters and centrality measures used to approximate the global functional significance of a node in network regulation [16,60].

### 4.6. Betweenness Centrality

Betweenness centrality quantifies how quickly a node can communicate with other pairs of nodes within the network. This nodal measure, denoted as C_B_(n), is computed by tallying the number of shortest paths that traverse through a specific node u (Equation (1)).

(1) $$C_{B}\left(n\right)=\sum_{v\in w\neq u}\frac{\sigma_{vw}\left(n\right)}{\sigma_{vw}}$$

where $\sigma_{vw}$ represents the shortest distance between node v and node w.

It reflects a node’s ability to leverage the information flow across the entire network, as well as its capacity to influence signal processing among other nodes within the network [16,58].

### 4.7. Closeness Centrality

Closeness centrality measures the proximity of a node to other nodes in a network, indicating how short a path is. The nodal measure $C_{C}\left(n\right)$ is calculated as the inverse of the sum of distances between the node of interest *u* and all other v ∈ V/n (Equation (2)).

(2) $$C_{C}\left(n\right)=\frac{1}{\sum_{v\neq n}d\left(n,v\right)}$$

A higher-value $C_{C}$ indicate faster propagation of information in the network.

This parameter depicts the speed of information distribution within a network, from one node to other connected nodes [61].

### 4.8. ROC Curve Analyses

A ROC (receiver operating characteristic) curve for hub genes was calculated using logistic regression analyses [62]. Logistic regression models provide straightforward interpretation of coefficients, making it easier to understand the influence of predictors on the outcome (e.g., biomarker status or disease prognosis). The ROC curve plots the True Positive Rate (Sensitivity) against the False Positive Rate (1 − Specificity) for all possible thresholds. Further, the AUC is computed as the area under this curve. A perfect classifier would have an AUC of 1.0, while a completely random classifier would have an AUC of 0.5.

$$True\;Positive\;Rate\;\left(TPR\right)=\frac{TP}{TP+FN}$$

where TP is True Positives and FN is False Negatives.

$$False\;Positive\;Rate\;\left(FPR\right)=\frac{FP}{FP+TN}$$

where FP is False Positives and TN is True Negatives.

The AUC is then computed by integrating the area under the ROC curve. It essentially measures the ability of the model to distinguish between classes.

### 4.9. Gene Set Variation Analysis (GSVA)

GSVA is a non-parametric and unsupervised technique for assessing the enrichment of transcriptome gene sets [63]. By thoroughly scoring the gene set of interest, GSVA transforms gene-level variations into pathway-level alterations, thereby evaluating the biological functions of samples. In this study, gene sets were obtained from the Molecular Signatures Database, and the GSVA algorithm was employed to score each gene set comprehensively, allowing for the evaluation of potential changes in biological function across different samples.

### 4.10. Identification of miRNAs and Functional Enrichment Analysis

The miRNAs for four key genes were retrieved using miRNet (www.mirnet.ca, accessed on 30 April 2024). The tool fetched miRNA–target interaction data from the miRTarBase [64], miRDB [65], and TargetScan [66] databases. The miRNA–mRNA interactions were utilized to generate the network using Cytoscape v3.6.1 (https://cytoscape.org, accessed on 28 May 2024). Significant biological pathways of candidate miRNAs were identified using the MIENTURNET platform [67]. The most statistically enriched GO terms were visualized using the ggplot2 visualization R package [68].

### 4.11. Drug–Genes Interaction

We predicted the target drugs of hub genes through the Drug–Gene Interaction Database (DGIdb) [69]. The DGIdb is a web resource that offers information on drug–gene interactions and druggable genes sourced from publications, databases, and other web-based platforms. The bar plots depicting interaction scores were generated using the ggplot2 package in R [68].

## 5. Conclusions

This study evaluates the network biology approach for screening potential biomarkers for HNSCC. We used the Network Analyzer and CytoHubba modules from Cytoscape to identify key regulator genes from the TCGA–HNSCC dataset. This study identified and analyzed four core genes, namely, *FN1*, *COL1A1*, *MMP9*, and *IL1A*, associated with the development and progression of HNSCC. These findings could enhance our understanding of the carcinogenesis process and provide indicators for prognosis and early disease detection. The enrichment of these genes has revealed associations with cellular communication, metabolic processes, and various biological regulations. Additionally, we identified significant deregulated miRNAs associated with these key genes, which were further analyzed to explore their connections with cancer pathways. In addition, the four key genes exhibited close interactions with both new types of anticancer agents and traditional chemotherapy drugs. However, these findings are based on bioinformatics analysis; therefore, experimental research is necessary to validate our results concerning the pathogenesis of head and neck cancer. This validation can provide the most accurate information for the prevention and therapy of head and neck cancer.

## Data Availability

All study data are included in this article.
